# Adalimumab in the management of psoriasis and psoriatic arthritis: Results from a Delphi investigation

**DOI:** 10.1515/rir-2024-0006

**Published:** 2024-03-31

**Authors:** Marco Matucci-Cerinic, Francesco Ciccia, Rosario Foti, Alessandro Giunta, Francesco Loconsole, Francesca Prignano, Rossana Scrivo, Giampiero Girolomoni

**Affiliations:** Department of Experimental and Clinical Medicine & Department of Geriatric Medicine, Division of Rheumatology AOUC, University of Florence, Italy and Unit of Immunology, Rheumatology Allergy and Rare Disease (UnIRAR), IRCCS San Raffaele, Milan, Italy; Department of Precision Medicine, University of Campania L. Vanvitelli, Naples, Italy; Unit of Immunology, Rheumatology Allergy and Rare Disease (UnIRAR), IRCCS San Raffaele, Milan, Italy; Department of Dermatology, University of Rome Tor Vergata, Rome, Italy; Department of Medicine Dermatology Section, University of Bari, Bari Italy; Department of Health Science, Section of Dermatology, University of Florence, Florence, Italy; Department of Clinical Internal, Anesthesiological and Cardiovascular Sciences, Rheumatology Unit, Sapienza University of Rome, Rome Italy; Department of Medicine, Section of Dermatology, University of Verona, Verona, Italy

**Keywords:** adalimumab, consensus, psoriasis, psoriatic arthritis, switch

## Abstract

**Background and Objectives:**

Psoriasis (PsO) and psoriatic arthritis (PsA) are often undertreated and require a multidisciplinary approach. In recent years, patent expiration has allowed the introduction of tumor necrosis factor inhibitor (anti-TNF) biosimilars, which have stimulated a significant increase in the use of biological therapies. This article reports the findings of a multidisciplinary approach to achieve a consensus on the use of adalimumab in patients with PsO or PsA.

**Methods:**

A voting panel of 36 Italian dermatologists and rheumatologists were chosen by eight Italian clinicians (the Board), to provide a consensus on the real-world management of PsO and PsA with adalimumab using the Delphi Method, comprising three survey rounds. Twelve statements were defined by the Board and submitted to the panel (rating scale 1–7).

**Results:**

Clinicians reached a wide consensus on the effectiveness (score 6–7: 67%) and long-term efficacy (6–7: 100%) of adalimumab in all clinical forms of PsO and PsA, including pediatric patients (6–7: 85%). Considering cost-effectiveness and safety, adalimumab is suggested as a first-line treatment in patients with enthesitis, predominant peripheral arthritis, axial involvement or associated inflammatory bowel disease (IBD) or uveitis. Adalimumab can be also considered after failure of etanercept (6–7: 94%).

**Conclusion:**

Results from this Delphi study clearly show an overall consensus on the use of adalimumab in the management of PsO and PsA, particularly as first-choice for specific subpopulations (uveitis, IBD, hidradenitis suppurativa). Considering the cost-effectiveness of biosimilars within Italy, adalimumab may represent an effective and safe first-line treatment for patients with moderate-to-severe PsO or PsA, and a valid choice for switching after failure.

## Introduction

Immune-mediated inflammatory diseases (IMIDs) are chronic disorders sharing common epidemiological and genetic features, and underlying pathogenetic pathways.^[1,2]^ Indeed, evidence suggests that multiple IMIDs may coexist in the same patient.^[3]^ In the first year of disease, peripheral arthritis may occur in about 12% of patients with inflammatory bowel disease (IBD).^[4]^ These patients are also prone to develop psoriasis (PsO) or rheumatoid arthritis (RA).^[5]^ In women with PsO and psoriatic arthritis (PsA), the former is associated with a significantly increased risk of Crohn’s disease.^[6]^

PsA occurs in 0.04%–1% of the general population and between 20%–40% of PsO patients.[7,8] Metabolic, cardiovascular,^[9]^ and psychological comorbidities overlap in PsO, with a significant impact on quality of life^[10]^ and increased mortality.^[11]^ The heterogeneous clinical presentation of PsA and PsO represents a clinical challenge when choosing a suitable therapy, in particular when treating concurrent comorbidities.^[12]^ Undertreatment is also significant,^[13,14]^ where up to 24% of patients with moderate-to-severe PsO are not treated, and 30% are only receiving topical therapy, which may not control the disease.^[13]^ Similarly, PsA is a heterogeneous and potentially severe disease, which may require a multidisciplinary approach to treatment.^[15]^

In the last two decades, the number of disease-modifying antirheumatic drugs (DMARDs) has significantly increased.^[16]^ The introduction of biologic DMARDs, such as tumor necrosis factor (TNF) inhibitors has greatly improved patients’ quality of life,^[1]^ with anti-TNFs often prescribed as first-line treatment for PsO and/or PsA.^[15,17]^ Guidelines also recommend anti-TNFs when there is an insufficient response to nonsteroidal anti-inflammatory drugs (NSAIDs) or local glucocorticoid injections.^[1,15,18]^ In PsA, a biologic DMARD is recommended after an inadequate response to at least one conventional synthetic DMARD, while an interleukin (IL)-17 or IL-12/23 inhibitor may be preferred when there is significant skin involvement.^[15]^ However, IL-17 inhibitor treatment has been associated with exacerbations of and new onset IBD or colitis. Fortunately, the cessation of IL-17 inhibitors and initiation of an alternative treatment (*e.g*., corticosteroids and anti-TNF therapy) can generally lead to clinical remission.^[19,20]^

The efficacy and tolerability of adalimumab have been demonstrated in PsA, plaque PsO, RA and IBD (including Crohn’s disease, ulcerative colitis, pediatric Crohn’s disease, and pediatric ulcerative colitis),^[21,22]^ Assessment of the clinical efficacy and cost-effectiveness of adalimumab has shown it to be a valid treatment choice in a large number of patients.^[1]^ Two Italian studies have demonstrated that, economically, adalimumab is below the threshold value for health care interventions for all its main indications, as it significantly reduces societal costs for RA, PsO and PsA, ankylosing spondylitis, and Crohn’s disease.^[23,24]^ The longterm safety profile of adalimumab has also been well established in multiple clinical trials,^[25]^ with infections being the most commonly reported serious adverse event, and in the real-world setting.^[26,27]^

It is generally accepted that patients with IMIDs should receive effective treatment as early as possible to prevent and limit organ damage, comorbidities, and the natural progression of the disease.^[28]^ Recent patent expiration has introduced numerous anti-TNF biosimilars, which have markedly increased the overall uptake of biological therapies for patients with PsO.^[29]^ Anti-TNF biosimilars provide cost reduction and increased patients’ access to biological treatment, positively influencing the course of their disease.^[1]^

The aim of the present work was to convene rheumatologists and dermatologists experienced in the use of anti-TNF agents, to achieve a consensus on the use of adalimumab in patients with PsO and/or PsA.

## Materials and Methods

A total of eight Italian clinicians (four rheumatologists and four dermatologists, hereafter referred to as the Board), with longstanding expertise in the treatment of PsA and PsO, gathered to reach a consensus on the management of both diseases with anti-TNF agents, by adopting the Delphi methodology.^[28, 29, 30, 31]^ The Delphi method is a highly regarded approach which involves an iterative process, characterised by multiple rounds of voting, to ascertain consensus on clinical matters in healthcare where there is limited guidance and/or a scarcity of evidence.^[30]^

In the present study, the methodology was stratified into four phases: [1] the Board identified 12 statements lacking clinical consensus and developed a Delphi questionnaire (Table 1), [2] the questionnaire was then submitted to a panel of 36 Italian clinical experts in the field of PsO and PsA through an online platform for a first Delphi round. The voting panel were chosen by the Board members, who nominated 4–5 collaborators or colleagues, each operating in distinct geographic regions (*e.g*., North-East, North-West). Geographical criteria were adhered to, ensuring homogeneous coverage of the Italian national territory. The panel were then asked to express their agreement or disagreement on each item using a Likert-type scale from 1 to 7 (1 = strongly disagree to 7 = strongly agree) for a maximum of three rounds. [3] Responses were collected and analysed. [4] Common and conflicting viewpoints were identified. At the end of Round 1, the median value, the 25th (Q1) and 75th (Q3) percentiles and the interquartile range (IQR) of each statement were calculated.

**Table 1:** Delphi consensus statements

NO	Statement
S1	A synergistic collaboration between dermatologists and rheumatologists may be very important for a more comprehensive and personalized management of patients with psoriatic disease (PsA and PsO)
S2	Adalimumab is highly effective in all clinical forms of PsO (scalp, nails, palmoplantar, inverse, face)
S3	Pediatric patients (>= 4 years old) with moderate to severe PsO and eligible for a systemic therapy are good candidates for treatment with adalimumab
S4	Adalimumab has a long-term sustained effectiveness in patients with PsA and PsO
S5	Cost-effectiveness considerations are very relevant in the choice of the treatment for patients with PsO and PsA
S6	Based on effectiveness, safety and costs, a patient with PsO eligible for a systemic treatment is a good candidate for adalimumab biosimilar as first-line treament
S7	Patients with PsA and predominant enthesitis are good candidates for treatment with adalimumab
S8	Patients with PsA and predominant peripheral arthritis are good candidates for treatment with adalimumab
S9	Patients with PsA and predominant axial involvement are good candidates for treatment with adalimumab
S10	Adalimumab is a drug of choice when PsO and/or PsA are associated with uveitis or hidradenitis suppurativa
S11	Adalimumab is a drug of choice when PsA and/or PsO are associated with inflammatory bowel disease
S12	In patients with PsA and moderate skin involvement the switch to adalimumab can be considered after failure of etanercept

PsA, psoriatic arthritis; PsO, psoriasis; S, statement.

In Round 2, experts were asked to answer the same statements taking into account the IQR of each question, as an index of the responses of their colleagues. In case of a score outside the IQR, experts were required to provide a reason. The results of the first and second rounds and the reasons were discussed by the Board, focusing on the motivations/theses of those who responded outside the IQR. After discussing and commenting on the results of each of the 12 statements, the Board members re-formulated the statements.

**Figure 1:**
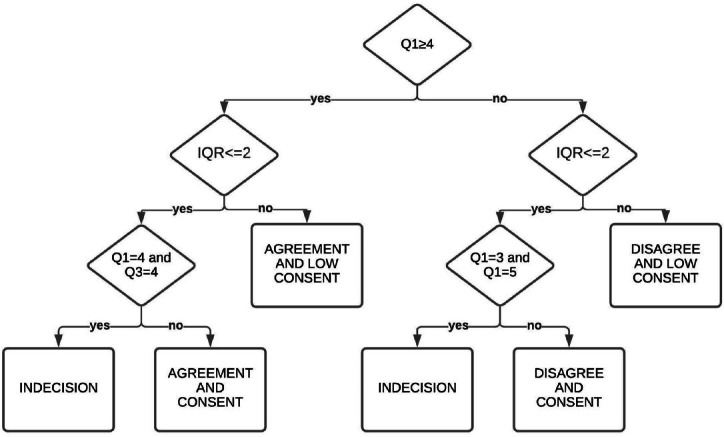
Flow diagram of the statement classification in terms of level of agreement or disagreement. IQR, interquartile range; Q, quartile.

In Round 3, the panel were invited to express their agreement/disagreement with the same statements considering the antitheses and the new IQRs. At this time, those who answered outside the IQR were asked to provide a new motivation. The statements were ranked based on the Q1 and the IQR (Figure 1). The “level of agreement or disagreement” achieved was measured according to the following criteria: a) agreement and consent (Q1 ⩾4, Q3 > 4 and IQRS2), b) agreement and low consent (Q1 ⩽4 and IQR⩽3), c) indecision (Q1= Q3= 4 or Q1= 3 and Q3= 5), d) disagree and consent (Q1 < 4 and IQR ⩽ 2 and Q1 ≠3 and Q1 #5) and e) disagree and low consent (Q1 < 4 and IQR⩾3).

### Statistical Analysis

Descriptive statistics (median and IQR) were used for the analysis of surveys in rounds. For each question and round the panel’s changes of opinion and related IQRs are reported, where the IQR is defined as the absolute value of the difference between Q3 and Q1, with smaller values indicating higher degrees of consensus. Statistical analyses were performed using Stata Version 16.1.

## Results

All 36 participants responded to Round 1, and 33 responded to the remaining two rounds. All statements reached a positive consensus, with variations in 29% of responses between the first and the second round, and in 17% of responses between the second and the third round. For the 4 statements with values outside the IQR (S6, S7, S9, and S12), the Board was asked to reclarify their statement to avoid confusion in the observations provided by the expert panel (Table 2).

**Table 2:** Thesis and antithesis motivation (in bracket the assigned score

No	Round	Thesis	Antithesis
S6	2	Analysis of costs, safety and effectiveness is not always in favor of a biosimilar (scored 4)	The statement of the board specifically refers to ADA biosimilars, while the objection pertains to biosimilars in general when compared with first-line agents
S7	2	I would consider, at least at the same level, an anti-IL-17 therapy in patients with enthesitis (scored 4)	We agree that both treatments are good candidates for the treatment of enthesitis. The question however was specifically related to ADA
S9	2	Only the MAXIMISE study with secukinumab has demonstrated clinical efficacy of a biologic drug in axial PsA (scored 3)	We agree that MAXIMISE is the only RCT on biotechnological drugs on axial PsA. However, TNF-blocking agents are currently recommended by GRAPPA and EULAR guidelines for the treatments of axial PsA.
S12	2	I personally don’t like cycling with anti-TNF (scored 1)	Switching among anti-TNF agents is a therapeutic option supported by scientific evidence
S12	3	We have now different biologic classes. Most of them are approved for both PsO and PsA. In my opinion, after the failure of an anti-TNF, switching to another class of biologics and changing the mechanism of action would be more desirable because anti-ILs have demonstrated superiority (scored 5)	
S12	3	I prefer to switch to another biologic (scored 3)	

ADA, adalimumab; IL, interleukin; RCT, randomized controlled trial; TNF, tumor necrosis factor; PsA, psoriatic arthritis; PsO, psoriasis.

### Consensus Statements

The final consensus for each item is summarized in Table 3.

**Table 3:** Statements and results of the Delphi consensus process

No	Round 1 (*n* = 36) median score (Q1-Q3: IQR)	Round 2 (*n* = 33) median score (Q1-Q3: IQR)	Round 3 (*n* = 33) median score (Q1-Q3: IQR)	Consensus degree
S1	7 (7-7: 0)	7 (7-7: 0)	7 (7-7: 0)	Agreement and consent
S2	5 (5-6: 1)	6 (5-6: 1)	6 (5-6: 1)	Agreement and consent
S3	6 (5-7: 2)	6 (5-7: 2)	6 (6-7: 1)	Agreement and consent
S4	6 (6-7: 1)	6 (6-7: 1)	6 (6-7: 1)	Agreement and consent
S5	6 (5-6.5: 1.5)	6 (5-7: 2)	6 (6-7: 1)	Agreement and consent
S6	6 (5-7: 2)	6 (6-7: 1)	6 (6-7: 1)	Agreement and consent
S7	6 (5-7: 2)	6 (5-7: 2)	6 (6-7: 1)	Agreement and consent
S8	6 (6-7: 1)	6 (6-7: 1)	6 (6-7: 1)	Agreement and consent
S9	6 (6-7: 1)	7 (6-7: 1)	7 (6-7: 1)	Agreement and consent
S10	7 (7-7: 0)	7 (7-7: 0)	7 (7-7: 0)	Agreement and consent
S11	7 (7-7: 0)	7 (7-7: 0)	7 (7-7: 0)	Agreement and consent
S12	6 (5-7: 2)	6 (6-7: 1)	6 (6-7: 1)	Agreement and consent

IQR, interquartile range; Q, quartile; S, statement.

**Statement S1. A synergistic collaboration between dermatologists and rheumatologists may be very important for a more comprehensive and personalized management of PsA and PsO patients**. This statement showed a high level of agreement in Round 1, with a median equal to 7. In Round 3, 97% of the experts gave 7, the maximum agreement score, with the remaining 3% (1 expert) assigning a score of 6. A 15% variation in answers was observed between the first and the second round, while only 1 expert (3%) changed his answer between the other rounds.

**Statement S2. Adalimumab is highly effective in all clinical forms of PsO (scalp, nails, palmoplantar, inverse, face)**. A median response equal to 5 was obtained in Round 1. In Round 2, all responses were between 5 and 6, while in Round 3, 33% of the experts gave a score equal to 5, 64% assigned a score of 6, and only one expert (3%) assigned the highest score. A 45% variation in answers was observed between the first and the second round, while 7 experts (21%) changed their answers between the other rounds.

**Statement S3. Pediatric patients (>= 4 years old) with moderate-to-severe PsO and eligible for a systemic therapy are good candidates for treatment with adalimumab**. The statement showed a high agreement from Round 1 (Table 3). In Round 3, 33% of experts assigned the maximum agreement score of 7. A variation in responses of 24% was observed between Round 1 and Round 2, and 27% between Round 2 and Round 3.

**Statement S4. Adalimumab has a long-term sustained effectiveness in patients with PsA and PsO**. A median score of 6 was achieved in Round 1. In Round 2, all responses were between 6 and 7. In Round 3, 30% of experts gave the maximum agreement score of 7, with the remaining 70% assigning a score of 6. A variation in responses of 24% was noted between Round 1 and Round 2, while three experts (9%) changed their answers in the other rounds.

**Statement S5. Cost-effectiveness considerations are very relevant in the choice of the treatment for patients with PsO and PsA**. A high agreement with a median of 6 was achieved in Round 1 and confirmed in Round 2. In Round 3, 33% of experts gave the maximum agreement score of 7, and 42% assigned a score of 6. Between Round 1 and Round 2, there was a variation in responses of 36%, while 33% of responses were modified by the experts between Round 2 and Round 3.

**Statement S6. Based on effectiveness, safety and costs, a patient with PsO eligible for a systemic treatment is a good candidate for adalimumab biosimilar as first-line treatment**. A high consensus with a median of 6 was reached in Round 1 and confirmed in Round 2. In Round 3, 39% of the experts gave the maximum agreement score of 7, and 61% gave a score of 6. Variations in responses of 36% and 39% were observed between Round 1 and Round 2, and between Round 2 and Round 3, respectively.

**Statement S7. Patients with PsA and predominant enthesitis are good candidates for treatment with adalimumab**. A high agreement with a median of 6 was achieved in Round 1 and confirmed in Round 2. All the answers ranged between 6 and 7 in Round 3, where 39% of the experts gave the maximum agreement score of 7, and 61% gave a score of 6. A variation of 27% in responses was observed between Round 1 and Round 2, while 6 experts (18%) changed their answers in the other rounds.

**Statement S8. Patients with PsA and predominant peripheral arthritis are good candidates for treatment with adalimumab**. A high agreement in Round 1 was achieved and confirmed in Round 2. The highest consensus was detected in Round 3, where 42% of the experts gave the maximum agreement score of 7, and 58% assigned a score of 6. A variation in responses was observed in 30% of responses between Round 1 and Round 2, while the response was modified by 3 experts (9%) in Round 3.

**Statement S9. Patients with PsA and predominant axial involvement are good candidates for treatment with adalimumab**. A high consensus of 6 was achieved in Round 1, which increased to a median of 7 in Round 2. In Round 3, 55% of experts gave the maximum score of 7, and 45% assigned a score of 6. There was a variation of 36% in responses between Round 2 and 3, while the answer was modified by 3 experts (9%) in Round 3.

**Statement S10. Adalimumab is a drug of choice when PsO and/or PsA are associated with uveitis or hidradenitis suppurativa**. All three rounds achieved the highest agreement with a median score of 7. While five experts (15%) changed their answers between Round 1 and Round 2.

**Statement S11. Adalimumab is a drug of choice when PsA and/or PsO are associated with IBD**. The highest median score of 7 was obtained in all rounds. A variation of 24% was seen in responses between Round 1 and 2.

**Statement S12. In patients with PsA and moderate skin involvement, the switch to adalimumab can be considered after failure of etanercept**. This statement received a high consensus among experts with a median score of 6 in Round 1. In Round 2, scores ranged between 6 and 7. In Round 3, 33% of the experts gave the maximum score of 7, and 61% gave a median score of 6. A variation of 36% in responses was detected between Round 1 and Round 2, while in Round 3, the answer was modified by 36% of the experts.

## Discussion

The results of this Delphi exercise clearly show that an overall, multidisciplinary consensus may be reached for the use of adalimumab in the management of PsO and PsA. The Delphi method is typically used in healthcare research to determine consensus in response to defined clinical questions.^[31]^ In this study the Delphi questionnaire received controlled feedback from a panel of experts in the field of PsO and PsA management. Moreover, the importance of collaboration between rheumatologists and dermatologists is unanimously accepted in Statement S1. In all statements, the Delphi procedure identified a high degree of consensus. Moreover, the statements are also widely supported in the literature, which further corroborates a satisfactory level of expertise, as well as a good level of communication between the centers. From a general point of view, the statements submitted to the panel have been shaped according to the following principles. Firstly, that the paradigm for the use of biologics in case of failure, intolerance, or contraindications to conventional synthetic DMARDs is getting stronger. Furthermore, in parts of the European Union where the use of biosimilars is characterised by an affordably low cost, their use as first-line treatment is moving progressively forward in light of their good long-term safety profile.^[32]^ Secondly, the association of a significant reduction in quality of life and ability to work with moderate-to-severe PsO and PsA^[33]^ may profit from a reduction in disease burden and the indirect costs of PsO.^[15,34]^ Moreover, adherence and patient satisfaction are higher with biologic agents when compared to conventional agents.^[35,36]^

In the present study, five statements in the Delphi questionnaire were based on the pathogenesis of IMIDs and the dys-regulation of inflammatory cytokines. The inhibition of TNF results in a down-regulation of the abnormal inflammatory pathways implicated in the pathogenesis and progression of IMIDs, and anti-TNF inhibitors including adalimumab may improve long-term patient outcomes by preventing the development of future damage and comorbidities.^[28]^

Among Delphi expert panel responses, adalimumab emerged as a drug with long-term sustained effectiveness in PsA and PsO. Moreover, the cost-effectiveness of adalimumab is a significant factor when considering treatment options for PsO and PsA. Therefore, the cumulative results of this study suggest that adalimumab offers a suitable first-line treatment for patients with PsO/PsA, offering an effective therapeutic option with an established long-term, risk/benefit profile. The panel also strongly agreed that adalimumab may be first choice for specific PsO and PsA subpopulations such as those with comorbid uveitis, IBD or hidradenitis suppurativa. The use of adalimumab in PsA patients with predominant enthesitis, and/ or peripheral arthritis, and/or axial involvement achieved a high agreement among the Board and expert panel.

In conclusion, the results of this Delphi exercise indicate the need to bridge the gap between patients’ expectations and physicians’ objectives, with a focus on improving the quality of life and personalized treatment of patients with PsO and PsA. Given the cost-effectiveness of biosimilars in Italy, adalimumab may represent an effective and safe first-line treatment for patients with moderate-to-severe PsO or PsA, as well as patients suffering from non-musculoskeletal symptoms affecting the gut (IBD) or the eyes (uveitis).
